# Effect of laparotomy on respiratory muscle activation pattern

**DOI:** 10.14814/phy2.12668

**Published:** 2016-01-05

**Authors:** Pritish Mondal, Mutasim Abu‐Hasan, Abhishek Saha, Teresa Pitts, Melanie Rose, Donald C. Bolser, Paul W. Davenport

**Affiliations:** ^1^ Department of PediatricsCollege of MedicinePennsylvania State UniversityPennsylvania; ^2^ Department of PediatricsUniversity of FloridaGainesvilleFlorida; ^3^ Department of StatisticsUniversity of FloridaGainesvilleFlorida; ^4^ Department of Neurological Surgery and Kentucky Spinal Cord Injury Research CenterUniversity of LouisvilleLouisvilleKentucky; ^5^ Department of Physiological SciencesUniversity of FloridaGainesvilleFlorida

**Keywords:** Diaphragm, hypercapnia, parasternal intercostal muscles, post‐inspiratory inspiratory activity PIIA, respiratory compensation

## Abstract

Muscular tone of the abdominal wall is important in maintaining transdiaphragmatic pressures and its loss can lead to decreased lung volumes. Patients who are status postlaparotomy are at risk of developing atelectasis. The compensatory role of respiratory muscle activity in postlaparotomy is not well studied. Normally, inspiratory muscles are active during inspiration and passive during expiration to allow for lung recoil. However, electrical activities of the inspiratory muscles continue during early expiratory phase to prevent rapid loss of lung volume. This activity is known as post‐inspiratory inspiratory activity (PIIA). In this study, we hypothesized that laparotomy will elicit an increase in PIIA, which is enhanced by respiratory chemical loading. Experiments were conducted in cats under three different conditions: intact abdomen (*n* = 3), open abdomen (*n* = 10), and post abdominal closure (*n* = 10) during eupnea and hypercapnia (10% CO_2_). Electromyography (EMG) activities of the diaphragm and parasternal muscles were recorded and peak EMG amplitude, PIIA time, and area under the curve were measured. Intraesophageal pressure was also obtained. PIIA was significantly higher under open abdominal conditions in comparison to intact abdomen during eupnea. Our data indicates that PIIA is increased during open abdomen and may be an important compensatory mechanism for altered respiratory mechanics induced by laparotomy. Also, PIIA remained elevated after abdominal closure. However, under hypercapnia, PIIA was significantly higher during intact abdomen in comparison to open abdomen, which is thought to be due to respiratory muscle compensation under chemical loading.

## Introduction

Stability and function of the respiratory system depends on the integrity of both the chest and the abdominal walls in order to maintain negative pleural pressure sufficient to inflate the lungs during inspiration and prevent their complete collapse during expiration. While the role of chest wall is obvious, the role of the abdominal wall integrity is not as extensively studied. It is already known that the abdominal wall is needed to primarily maintain a negative subdiaphragmatic pressure throughout normal respiration. This negative subdiaphragmatic pressure decreases inspiratory muscle loading during inspiration. It also counteracts lung recoil and helps to maintain lung expansion during expiration. Beecher et al. demonstrated that opening of peritoneal cavity leads to the loss of this subdiaphragmatic negative pressure resulting in loss of lung volume (Beecher 1933a,b). It requires about 72 h to restore this pressure after the anatomical closure of abdominal cavity.

Clinically, patients recovering from laparotomy suffer from inadequate oxygenation and ventilation in the immediate postoperative period due to decreased lung volume and lung collapse (i.e., atelectasis) (Lee et al. 1928). Similarly, children with congenital abdominal wall defect have breathing difficulties and often need ventilatory support (Panitch 2015).

While respiratory compensations are expected during loss of abdominal wall integrity, the exact mechanism is largely unknown. Previous work has suggested that inspiratory muscle activation pattern changes may provide the respiratory compensation necessary to sustain ventilation (Gandevia et al. 1999). We investigated this possible compensatory role of the respiratory muscles, namely the possibility of an increase in peak inspiratory muscle activity during inspiration or an increase in inspiratory muscle braking during expiration.

In the normal respiratory cycle, inspiration is shorter than expiration. The inspiratory:expiratory time ratio (I:E) varies from 1:2 to 1:3 (Strauss‐Blasche et al. 2000). Inspiration is an active process, requiring activation of the diaphragm the principal inspiratory muscle while expiration is mostly passive and driven by lung recoil. Even though inspiratory muscles relax during most of the expiratory phase, the electrical activity of the inspiratory muscles does not abruptly end at the end of mechanical inspiration, but continues in the early deflation phase of expiration. This phenomenon of inspiratory muscle activity during the early expiratory phase is known as inspiratory braking with inspiratory muscle activity referred to as Post‐Inspiratory Inspiratory Activity (PIIA) as described by Gautier et al. (1973). The role of PIIA counteracts the lung recoil deflation force and decreases the rate of lung deflation during expiration.

We posit that the loss of negative subdiaphragmatic pressure during open abdomen is more detrimental during expiration than inspiration leading to the loss of lung volume observed clinically and experimentally (Ainsworth et al. 1989). Since PIIA brakes deflation, it is likely to be an important compensatory mechanism when subdiaphragmatic, abdominal pressures are compromised by laparotomy. Therefore, we hypothesize that PIIA to be augmented in the open abdomen condition to compensate for the change in subdiaphragmatic pressure. Our experiments were designed to determine whether laparotomy influences the overall inspiratory muscle activation pattern and/or isolated PIIA. Since the negative subdiaphragmatic pressure does not immediately return to normal after abdominal closure, it was also important to observe whether the respiratory muscle activation compensatory pattern persists after the abdominal closure. In this study, respiratory muscle activity pattern with hypercapnic challenge was also determined. We further hypothesized that: (1) laparotomy (open abdomen) will elicit an isolated increase in PIIA; (2) PIIA changes persist even after immediate closure of abdomen; and (3) PIIA will be exacerbated with respiratory chemical load like hypercapnia.

## Materials and Methods

### Procedures

The experiments were done following the University of Florida Institutional Animal Care and Use Committee (IACUC) rules and the committee approval of the protocol. Experiments were performed on 10 adult male cats. Seven cats underwent respiratory muscle activity measurements during open abdomen (laparotomy) and post closure. Three animals had the same measurements during intact abdomen (before laparotomy), open abdomen, and post closure.

All animals were anesthetized with sodium pentobarbital (35 mg/kg i.v.) and subsequent doses were given as needed. A tracheostomy incision was made between fifth and sixth tracheal rings and a cannula inserted to access the airways, continuously monitor end‐tidal CO_2_ (ETCO_2_) and to deliver CO_2_ (10%) during the hypercapnic challenge. The animals were spontaneously breathing throughout the experiment. The right femoral artery was cannulated and used to monitor the arterial blood gases. Fluids were supplemented through the right femoral vein catheter. A balloon catheter was placed in the lower third of the esophagus to record esophageal pressure changes (PES) as a measure of intrathoracic pressure.

The seven animals that were studied with only the open abdomen and surgically closed abdomen conditions underwent midline laparotomy from xiphisternum to umbilicus (an average of 12 cm incision). Fine‐wire electrodes (insulated) were placed into the diaphragm, left parasternal intercostal (L‐PS), and right parasternal intercostal (R‐PS) muscles to record EMG activities using Basmajian and Stecko technique (Basmajian and Stecko 1962). The EMG signal was amplified, bandpass filtered (300–5000 Hz), digitized (2000 Hz), and recorded with a computer signal processing system (Cambridge Electronic Design, Model 1401). The three animals that were studied prelaparotomy, abdomen open, and abdomen surgically closed had the diaphragm electrodes inserted transcutaneously by placing the electrodes below the intercostal arch cranially into the diaphragm. Diaphragmatic electrode placement in these animals was confirmed when the abdomen was opened by the laparotomy. The laparotomy incision was closed with interlocking suture and adhesive applied along the tissue incision edges to ensure that the abdomen return to a completely closed cavity.

### Experimental protocol

Electromyographic (EMG) activity of respiratory muscles was recorded during intact abdomen (before laparotomy, *n* = 3), with the abdomen open after laparotomy (*n* = 10) and after closure of the abdomen (*n* = 10). When the animal was surgically prepared, 30 min was allowed to elapse before recordings were obtained. An arterial blood gas sample was obtained prior to the recording. EMG and PES signals were recorded for 15 min with the animal intact (*n* = 3). The animal was then exposed to 10% CO_2_, balance oxygen. After 5 min, hypercapnia, EMG, and PES were recorded for 5 min and then an arterial blood gas sample obtained. The animal was returned to room air and recovered from the hypercapnic challenge for at least 15 min. The laparotomy was then performed (*n* = 10) and the animal allowed 15 min to stabilize from the surgery. The EMG and PES signals were then recorded during eupneic room air breathing for 15 min. The animals were again presented the hypercapnic challenge trial. After recovery from the hypercapnic challenge, the laparotomy was closed. A 60 min waiting period was allowed post closure before the eupneic breathing and hypercapnic trials were repeated for the closed abdomen condition.

### Electromyographic signal analysis

All raw EMG signals from respiratory muscles (diaphragm, right parasternal, and left parasternal) were rectified and integrated (100 msec time constant) for analysis. Spike2 software was used for analysis of the data recordings. R and SPSS software were used for the statistical analysis.

In order to define PIIA and peak inspiratory muscle activity, it was necessary to define inspiration and expiration using PES signal. The lowest (most negative) esophageal pressure point was used to define the end of inspiration. The initial decrease in esophageal pressure was used to define onset of inspiration.

**Figure 1:**
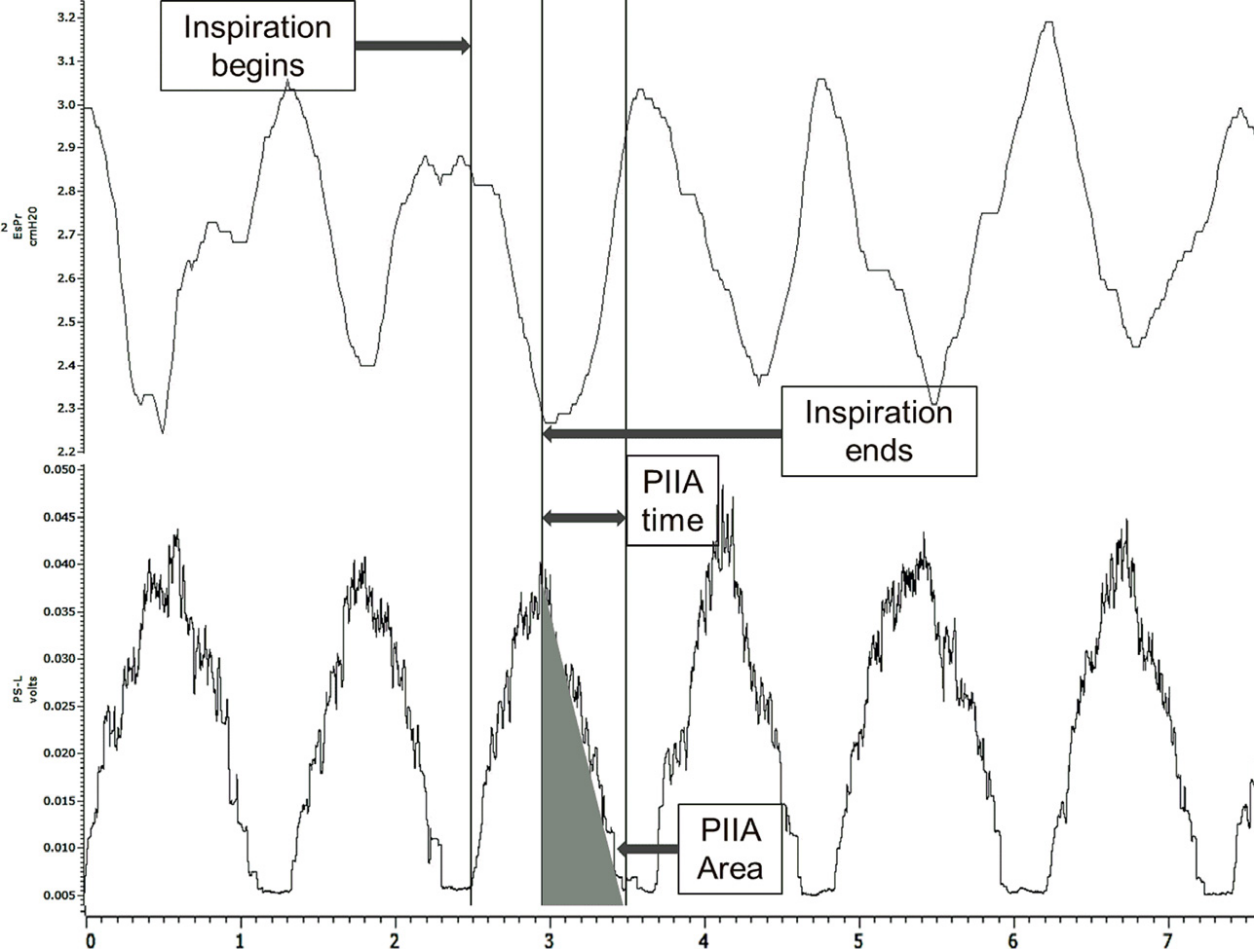
Diagram depicting esophageal pressure tracing (top) and left parasternal muscle EMG activity (bottom). Both are used to define PIIA time and area as demonstrated.

Inspiratory muscle peak EMG activity was identified as the highest integrated EMG activity. PIIA was identified as the EMG activity from the point of end of inspiration until EMG activity reaches baseline (Fig. 1). PIIA time and area under the curve (PIIA area) were then calculated. Since the influence of overall inspiratory muscle activity on the same respiratory cycle PIIA magnitude was uncertain, a parameter for PIIA was selected which should reflect isolated PIIA activity. Therefore, the ratio of PIIA time over total inspiratory EMG time (PIIA time ratio) and PIIA area over total inspiratory EMG area (PIIA area ratio) were used to compare between different experimental conditions.

Measurements from five consecutive breaths were analyzed for each of the six experimental conditions (intact eupnea, intact hypercapnia, open eupnea, open hypercapnia, post closure eupnea, and post closure hypercapnia) for each animal. A two‐way ANOVA model was fit for the six conditions.

$$y_{1}^{ijk}=\theta +\lambda_{s}^{ijk}+\mu_{\mathrm{abdomen}}^{ijk}+\epsilon_{1}^{ijk}$$

Where *i* is state of breathing (eupnea vs. hypercapnia), *j* denoted different muscles, and *k* represented different measurements (e.g., PIIA area ratio or PES). $y_{1}^{ijk}$ were calculated separately for peak amplitude, PIIA area, PIIA time, and PIIA ratios (where *l* represented 1,2,3,4,5 observations). On the right hand of the model, {$\epsilon_{1}^{ijk}$} denoted independent and identically distributed normal errors, {$\lambda_{s}^{ijk}$} were the effects for the animals (*s* = 1,2,…,10), and {$\mu_{\mathrm{abdomen}}^{ijk}$} were the effects for the three abdominal conditions. Since the effects corresponding to individual subjects were included, normalization of the data was not necessary. However, for between animal comparisons, regressions with the normalized data were performed and the multiple correlation coefficients calculated was low. The *P* values less than 0.05 was considered significant (Tables 1, 2, 3, 4, 5).

**Table 1:** Eupneic breathing (*n* = 3)

	Intact	Open	Intact versus Open
PIIA area			
Diaphragm	0.003 ± 0.00	0.005 ± 0.00	*P* < 0.001**
Left PS	0.008 ± 0.00	0.012 ± 0.00	*P* < 0.001**
Right PS	0.007 ± 0.00	0.015 ± 0.00	*P* < 0.001**
PIIA time			
Diaphragm	0.26 + 0.02	0.47 + 0.02	*P* < 0.001**
Left PS	0.38 + 0.23	0.45 + 0.23	*P* = 0.08
Right PS	0.32 + 0.03	0.56 + 0.03	*P* < 0.001**
PIIA area/Total inspiratory area			
Diaphragm	0.169 ± 0.04	0.454 ± 0.05	*P* < 0.001**
Left PS	0.28 ± 0.03	0.398 ± 0.02	*P* < 0.001**
Right PS	0.265 ± 0.02	0.408 ± 0.03	*P* < 0.001**
PIIA time/Total inspiratory time			
Diaphragm	0.208 ± 0.02	0.388 ± 0.03	*P* < 0.001**
Left PS	0.28 ± 0.04	0.361 ± 0.02	*P* = 0.01*
Right PS	0.249 ± 0.03	0.415 ± 0.03	*P* < 0.001**
Delta Pes	1.85 ± 0.21	1.91 ± 0.09	*P* = 0.75
Peak amplitude			
Diaphragm	0.005 ± 0.00	0.004 ± 0.00	*P* = 0.1
Left PS	0.034 ± 0.01	0.044 ± 0.01	*P* = 0.003*
Right PS	0.037 ± 0.01	0.030 ± 0.01	*P* = 0.187

Comparison between intact versus open abdominal trials under eupneic conditions. The numbers represent mean ± SEM. *denotes *P*‐value <0.05, **indicates *P*‐value <0.001.

**Table 2:** Hypercapnic breathing (*n* = 3)

	Intact	Open	Intact versus Open
PIIA area			
Diaphragm	0.006 ± 0.00	0.005 ± 0.00	*P* < 0.001**
Left PS	0.26 ± 0.00	0.21 ± 0.00	*P* < 0.001**
Right PS	0.03 ± 0.00	0.018 ± 0.00	*P* < 0.001**
PIIA time			
Diaphragm	0.41 + 0.02	0.22 + 0.02	*P* < 0.001**
Left PS	0.47 + 0.01	0.40 + 0.01	*P* < 0.001**
Right PS	0.49 + 0.03	0.37 + 0.03	*P* < 0.001**
PIIA area/Total inspiratory area			
Diaphragm	0.348 ± 0.05	0.295 ± 0.02	*P* = 0.014*
Left PS	0.372 ± 0.03	0.375 ± 0.02	*P* = 0.89
Right PS	0.379 ± 0.03	0.352 ± 0.02	*P* = 0.281
PIIA time/Total inspiratory time			
Diaphragm	0.328 ± 0.05	0.236 ± 0.01	*P* = 0.009*
Left PS	0.379 ± 0.03	0.359 ± 0.01	*P* = 0.4
Right PS	0.388 ± 0.03	0.337 ± 0.02	*P* = 0.063
Delta Pes	10.5 ± 0.92	14.09 ± 0.72	*P* = 0.003*
Peak amplitude			
Diaphragm	0.030 ± 0.01	0.028 ± 0.01	*P* = 0.189
Left PS	0.076 ± 0.01	0.096 ± 0.01	*P* = 0.003*
Right PS	0.116 ± 0.01	0.104 ± 0.02	*P* = 0.384

Comparison between intact versus open abdominal trials under hypercapnic conditions. The numbers represent mean ± SEM. *denotes *P*‐value <0.05, **indicates *P*‐value <0.001.

**Table 3:** Eupneic breathing (*n* = 10)

	Open	Closed	Open versus Closed
PIIA area			
Diaphragm	0.004 ± 0.00	0.005 ± 0.00	*P* < 0.001**
Left PS	0.019 ± 0.00	0.022 ± 0.00	*P* = 0.39
Right PS	0.009 ± 0.00	0.01 ± 0.00	*P* = 0.78
PIIA time			
Diaphragm	0.5 ± 0.02	0.37 ± 0.02	*P* < 0.001**
Left PS	0.73 ± 0.02	0.75 ± 0.02	*P* = 1
Right PS	0.77 ± 0.02	0.75 ± 0.02	*P* = 1
PIIA area/Total inspiratory area			
Diaphragm	0.361 ± 0.02	0.323 ± 0.02	*P* = 0.10
Left PS	0.398 ± 0.01	0.438 ± 0.03	*P* = 0.11
Right PS	0.393 ± 0.02	0.425 ± 0.02	*P* = 0.12
PIIA time/Total inspiratory time			
Diaphragm	0.331 ± 0.02	0.308 ± 0.02	*P* = 0.31
Left PS	0.342 ± 0.02	0.404 ± 0.02	*P* = 0.03*
Right PS	0.422 ± 0.02	0.442 ± 0.02	*P* = 0.26
Delta Pes	1.94 ± 0.11	2.08 ± 0.14	*P* = 0.2
Peak amplitude			
Diaphragm	0.013 ± 0.00	0.012 ± 0.002	*P* = 1
Left PS	0.04 ± 0.00	0.039 ± 0.00	*P* = 0.74
Right PS	0.019 ± 0.00	0.018 ± 0.00	*P* = 0.63

Comparison between open versus closed abdominal trials under eupneic breathing. Measurements are presented as mean ± SEM. *indicates *P*‐value <0.05, **indicates *P*‐value <0.001.

**Table 4:** Hypercapnic breathing (*n* = 10)

	Open	Closed	Open versus Closed
PIIA area			
Diaphragm	0.007 ± 0.00	0.009 ± 0.00	*P* = 0.002*
Left PS	0.024 ± 0.00	0.023 ± 0.00	*P* = 1
Right PS	0.012 ± 0.00	0.013 ± 0.00	*P* = 0.35
PIIA time			
Diaphragm	0.33 + 0.01	0.32 + 0.01	*P* = 0.89
Left PS	0.48 + 0.01	0.48 + 0.01	*P* = 1
Right PS	0.43 + 0.01	0.48 + 0.01	*P* = 0.002*
PIIA area/Total inspiratory area			
Diaphragm	0.3 ± 0.01	0.304 ± 0.01	*P* = 0.81
Left PS	0.354 ± 0.01	0.359 ± 0.01	*P* = 0.55
Right PS	0.351 ± 0.01	0.36 ± 0.011	*P* = 0.48
PIIA time/Total inspiratory time			
Diaphragm	0.305 ± 0.01	0.287 ± 0.01	*P* = 0.14
Left PS	0.383 ± 0.02	0.352 ± 0.01	*P* = 0.01*
Right PS	0.367 ± 0.01	0.388 ± 0.01	*P* = 0.15
Delta Pes	20.6 ± 1.51	17.4 ± 1.14	*P* = 0.10
Peak amplitude			
Diaphragm	0.065 ± 0.01	0.046 ± 0.01	*P* < 0.001**
Left PS	0.121 ± 0.01	0.111 ± 0.01	*P* = 0.08
Right PS	0.071 ± 0.01	0.0729 ± 0.01	*P* = 0.46

Comparison between open versus closed abdominal trials under hypercapnic breathing. The numbers represent mean ± SEM. *denotes *P*‐value <0.05, **indicates *P*‐value <0.001.

**Table 5:** Eupnea (E) versus Hypercapnia (H)

	Intact	Open	Closed
PIIA area			
Diaphragm	H > E (*P* < 0.001)**	H > E (*P* < 0.001)**	H > E (*P* < 0.001)**
Left PS	H > E (*P* < 0.001)**	H > E (*P* = 0.002)*	*P* = 0.16
Right PS	H > E (*P* < 0.001)**	H > E (*P* < 0.001)**	*P* = 0.55
PIIA time			
Diaphragm	H > E (*P* = 0.003)*	E > H (*P* < 0.001)**	E > H (*P* = 0.048)*
Left PS	H > E (*P* = 0.006)*	E > H (*P* < 0.001)**	E > H (*P* < 0.001)**
Right PS	H > E (*P* < 0.001)**	E > H (*P* < 0.001)**	E > H (*P* < 0.001)**
PIIA area/Total inspiratory area			
Diaphragm	H > E (*P* = 0.003)*	E > H (*P* = 0.003)*	*P* = 0.35
Left PS	H > E (*P* = 0.003)*	E > H (*P* = 0.001)**	E > H (*P* < 0.001)**
Right PS	H > > E (*P* < 0.001)**	E > H (*P* = 0.009)*	E > H (*P* = 0.001)*
PIIA time/Total inspiratory time			
Diaphragm	H > E (*P* = 0.008)**	*P* = 0.22	*P* = 0.26
Left PS	H > E (*P* = 0.004)*	H > E (*P* = 0.006)*	E > H (*P* = 0.001)**
Right PS	H > E (*P* < 0.001)**	E > H (*P* < 0.001)**	E > H (*P* < 0.001)**
Delta Pes	H > E (*P* < 0.001)**	H > E (*P* < 0.001)**	H > E (*P* < 0.001)**
Peak amplitude			
Diaphragm	H > E (*P* < 0.001)**	H > E (*P* < 0.001)**	H > E (*P* < 0.001)**
Left PS	H > E (*P* < 0.001)**	H > E (*P* < 0.001)**	H > E (*P* < 0.001)**
Right PS	H > E (*P* < 0.001)**	H > E (*P* < 0.001)**	H > E (*P* < 0.001)**

Table comparing eupnea versus hypercapnia for all experimental conditions (intact, open and closed abdomen). *denotes *P*‐value <0.05, **indicates *P*‐value<0.001.

## Results

### Intact versus open abdomen under eupnea

**Figure 2:**
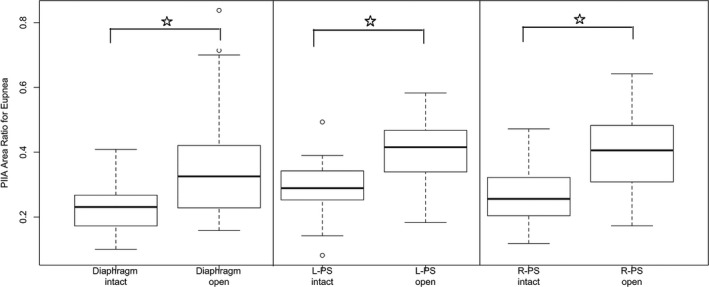
Post‐inspiratory inspiratory activity (PIIA) area ratio during eupneic breathing under intact versus open abdominal conditions showing significant increase in PIIA area ratio post laparotomy (*P* < 0.05). (○ represent outlier points, error bars represent 1.5 × IQR or range of variations above the third quartile and 1.5 × IQR below the first quartile, respectively).

**Figure 3:**
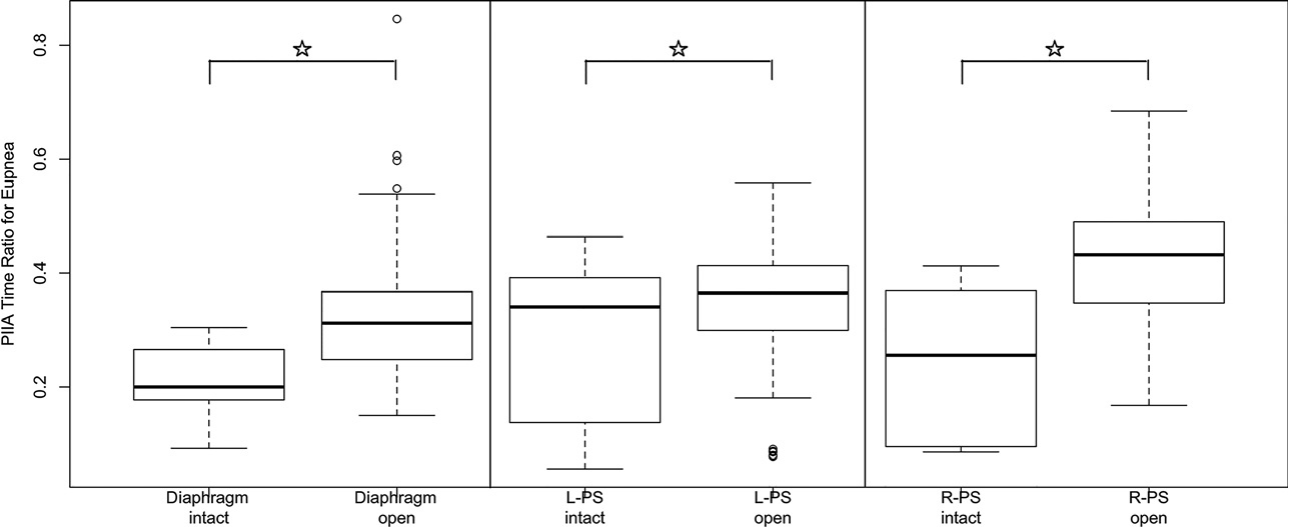
Post‐inspiratory inspiratory activity (PIIA) time ratio during eupnea under intact versus open abdominal conditions showing significant increase in PIIA time ratio after laparotomy. (○ represents outlier points, error bars represent 1.5 × IQR or range of variations above the third quartile and 1.5 × IQR below the first quartile, respectively).

Under eupneic conditions, PIIA time and PIIA area were significantly increased after opening the abdomen compared to intact abdomen in all muscle groups except of left PS. Also, PIIA time and area ratios were significantly increased after opening the abdomen compared to intact abdomen in all three muscle groups (Figs. 2, 3).

On the other hand, there was no significant difference in delta PES between intact abdomen and open abdomen conditions. Peak amplitude was increased during open abdomen in L‐PS muscles only (Table 1).

### Intact versus open abdomen under hypercapnia

Under hypercapnia, PIIA time and PIIA area were significantly decreased after laparotomy compared to intact abdomen in all three muscles. However, PIIA area and time ratios decreased significantly during postlaparotomy trials in diaphragm only, while both parasternals did not show any significant differences.

Delta PES was significantly increased postlaparotomy under hypercapnia. Peak amplitude was increased in L‐PS only after opening of abdomen (Table 2).

### Open versus closed abdomen under eupnea

There was no significant change in the PIIA time or PIIA area after the abdomen was closed in both parasternals. However, in the diaphragm, PIIA time and PIIA area were decreased significantly after abdominal closure.

**Figure 4:**
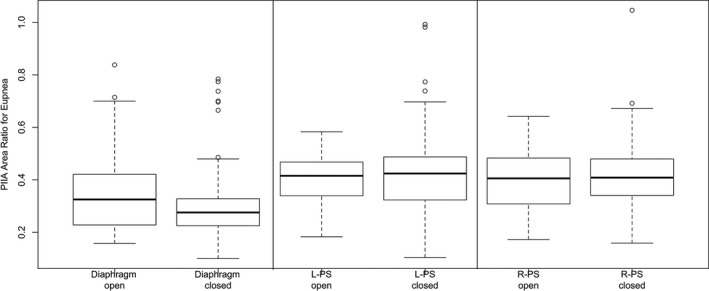
Post‐inspiratory inspiratory activity (PIIA) area ratio during open versus closed abdominal conditions under eupneic breathing shows no significant change (○ represent outlier points, error bars represent 1.5 × IQR or range of variations above the third quartile and 1.5 × IQR below the first quartile, respectively).

**Figure 5:**
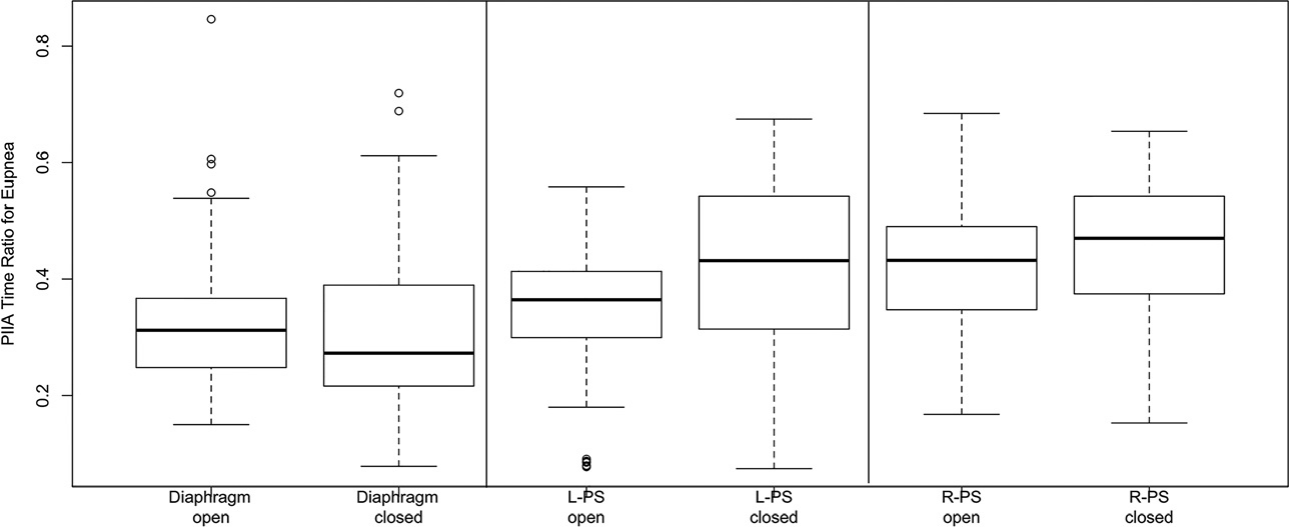
Post‐inspiratory inspiratory activity (PIIA) time ratio during eupneic breathing under open versus closed abdominal conditions showing no change in between the conditions (○ represent outlier points, error bars represent 1.5 × IQR or range of variations above the third quartile and 1.5 × IQR below the first quartile, respectively).

Post‐inspiratory inspiratory activity (PIIA) area ratio remained unchanged after closure of abdomen in all muscle groups (Fig. 4). Also, there was no significant difference in PIIA time ratio in the diaphragm and R‐PS (Fig. 5). However, PIIA time ratio was increased after abdominal closure in L‐PS (Table 3).

There was no significant difference in delta PES between open abdomen and closed abdomen under eupnea. Similarly, the peak amplitude was unchanged after the abdomen was closed in all muscle groups (Table 3).

### Open versus closed abdomen under hypercapnia

Post‐inspiratory inspiratory activity (PIIA) time remained unchanged in the diaphragm and the L‐PS after closing the abdomen, but in the R‐PS muscle, PIIA time was significantly increased. PIIA areas was also unchanged after abdominal closure in both parasternal, but increased in the diaphragm only. In all three muscles, PIIA area and time ratios remained unchanged after abdominal closure, except for the L‐PS where PIIA time ratio was decreased.

During hypercapnia, there was no significant difference in delta PES between open abdomen and closed abdomen. In both parasternals, there were no significant changes in peak amplitude. However, peak amplitude decreased significantly after abdominal closure in the diaphragm (Table 4).

### Eupnea versus hypercapnia under all abdominal conditions

When the abdomen was intact, PIIA time, PIIA area, PIIA time, and area ratios were all significantly higher under hypercapnia compared to eupnea in all muscle groups, which was consistent with the third hypothesis. However, during open abdomen as well as closed abdomen, a reverse trend was observed where PIIA time, PIIA time, and area ratios were mostly decreased under hypercapnia compared to eupnea except for the diaphragm where there was no significant difference. No consistent pattern was found with regards to PIIA area.

Esophageal pressure changes (PES) and peak amplitude increased consistently under hypercapnia in comparison to similar eupneic conditions under all three muscles (Table 5).

## Discussion

In this project, we investigated the effect of disrupting the abdominal wall integrity (i.e., laparotomy) on the respiratory muscle activity and specifically on inspiratory muscle breaking with post‐inspiratory inspiratory activity. Our results demonstrated that PIIA area and time ratios were significantly higher during open abdomen compared to intact abdomen. These results support our hypothesis that laparotomy related loss of abdominal pressure elicited a respiratory muscle motor pattern compensatory response with an increase in PIIA. Furthermore, PIIA time and area ratios did not differ between open and post closed abdomen, which supports our hypothesis that PIIA changes persist even after immediate closure of abdomen due to the time required to restore the thoracoabdominal pressure gradient. PIIA was described by Gautier et al. as inspiratory muscle activity that continues into the mechanical expiratory deflation phase after termination of tidal inspiratory airflow. PIIA is thought to be produced by the respiratory motor neurons (Gautier et al. 1973). These inspiratory neurons were described by Richter et al., as late inspiratory, which fire during the early expiratory period in phase with phrenic nerve discharge and generate inspiratory braking (Richter 1982).

Post‐inspiratory inspiratory activity (PIIA) plays a role as a protective braking mechanism, which prevents rapid loss of lung volume during expiration by counteracting the respiratory system recoil and the associated rapid decline in intrathoracic pressure. PIIA can also reduce the duration of the passive expiratory phase and may maintain higher functional residual capacity (FRC) (Remmers and Bartlett 1977; Easton et al. 1989). During open abdomen, there is a loss of the subdiaphragmatic pressure, which can lead to rapid loss of volume during expiration. An increase in PIIA braking is therefore needed to counteract this loss. To the best of our knowledge, no previous work has tested this hypothesis and evaluated the role of PIIA in open abdominal conditions.

**Figure 6:**
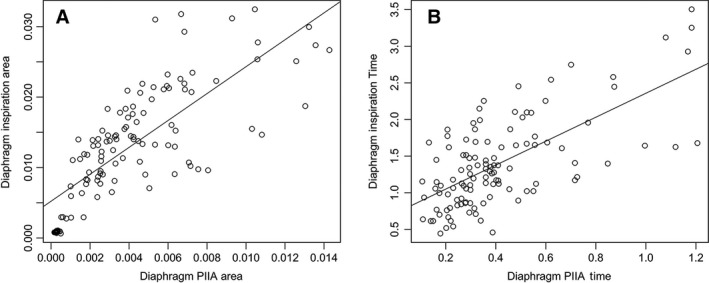
Regression variable plot between (A) Diaphragm total Inspiratory area and Diaphragm PIIA area under eupneic breathing (Pearson correlation 0.77, *P* < 0.05*) (B) Diaphragm Inspiratory time and Diaphragm PIIA time under eupnea (Pearson correlation 0.67, *P* < 0.05*) showing significant correlation.

**Figure 7:**
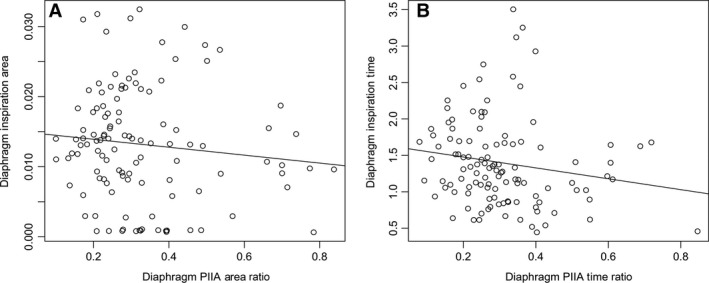
Regression variable plot between (A) Diaphragm total inspiratory area and Diaphragm PIIA area ratio (Pearson correlation −0.11) (B) Diaphragm Inspiratory time and Diaphragm PIIA time ratio under eupnea (Pearson correlation −0.17) showing no correlation.

To measure the PIIA activity, we investigated both PIIA area and time. PIIA area is a representation of inspiratory braking since PIIA area integrates both time and amplitude components. More importantly, we also used PIIA time and area as a ratio of the total inspiratory time and area, respectively, in addition to using absolute values to compare between experimental conditions as has been used previously (Kosch et al. 1988; Easton et al. 1999). This use of ratios is based on our observation that PIIA time and area are strongly correlated with total inspiratory time and total inspiratory area (respectively) of the same breath (Fig. 6). So, increase in PIIA time and PIIA area could be a reflection of increased total inspiratory activity, thus of no individual significance. It was necessary to recognize a parameter for PIIA area/time, which was independent of total inspiratory area and time, respectively. Hence, the PIIA area and PIIA time ratios were measured to establish a specific increase in PIIA activity, which was independent of the total inspiratory muscle activity. Figure 7 shows no significant correlation between PIIA area/time ratio of diaphragm and total inspiratory time, and further corroborates our observations that PIIA time and area ratios are parameters that can be used in future investigations.

The significant increase in PIIA activity during open abdomen persisted even after abdominal closure suggesting that pressure and mechanical changes induced by opening the abdomen are not immediately reversed by closing the abdomen. Easton et al. reported that about 72 h is required to stabilize the breathing pattern post abdominal closure. In our experiments, PIIA did not change 1–3 h post closure conditions suggesting that immediate closure of the abdomen does not reestablish the subdiaphragmatic pressure gradient and supports the need for the extended postlaparotomy recovery time (Easton et al. 1989).

Citterio et al. described a positive correlation between diaphragm and parasternal PIIA time during both inspiration and post inspiratory phase. The motor pattern for both the diaphragm and parasternal intercostal muscles found in this study suggest that these muscles are coordinated to generate both inspiratory muscle drive and PIIA braking. The changes in PIIA found in all these muscles suggest a central neural origin of the motor pattern that compensates for the changes in thoracoabdominal mechanics during and after laparotomy (Citterio and Agostoni 1981).

**Figure 8:**
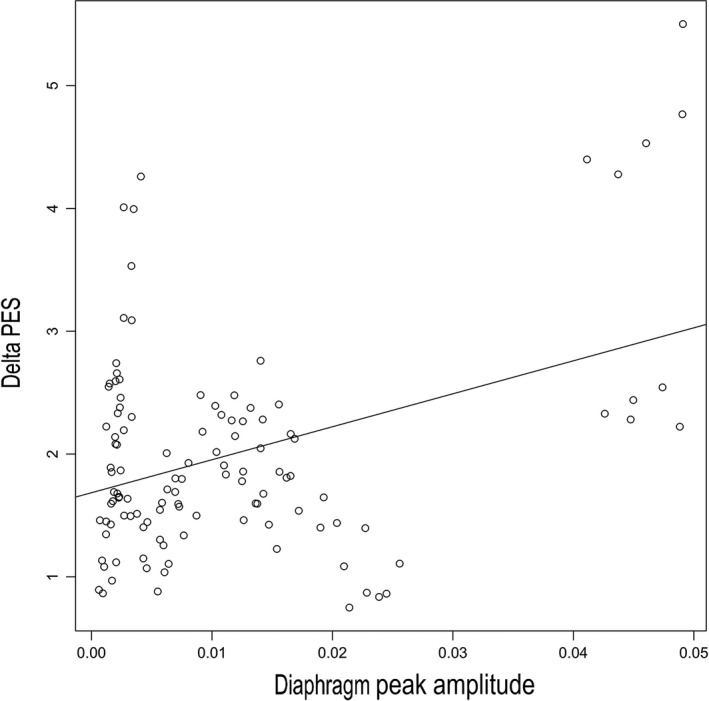
Regression variable plot between Diaphragm peak amplitude and Delta PES under eupneic breathing showing significant correlation (Pearson correlation 0.38, *P* < 0.05*).

Our experiments show unequivocally that diaphragm EMG activity was actually present during intact abdomen, laparotomy, and post abdominal closure and correlated well with esophageal pressure (Fig. 8). However, we did not find a consistent change in peak amplitude in all three muscle groups after laparotomy (Intact vs. open) (Table 1). Also, the peak amplitude was not different when open abdominal conditions were compared to closed under eupneic breathing. These findings suggest that the peak diaphragmatic muscle activity is less important than its inspiratory braking during an abdominal open condition. It is understandable that peak inspiratory muscle activity influence transdiaphragmatic pressure during inspiration as demonstrated by our finding of strong correlation between esophageal pressure and peak diaphragmatic EMG activity (Fig. 8). Previous work by Burgess et al. described a decrease in the function of the diaphragm in the immediate postoperative period after laparotomy in dogs (Burgess et al. 1984). Transdiaphragmatic pressure changes were used as a measure of diaphragm function. In addition, Ford et al. used transdiaphragmatic pressure to describe diaphragmatic function in human subjects (Ford et al. 1983). The use of transdiaphragmatic pressure as a measure for diaphragmatic muscle activity during open abdomen is influenced by multiple factors including subdiaphragmatic pressure, chest wall, and abdominal compliance as well as diaphragmatic muscle activity. In our experiments**,** intrathoracic pressure was measured by esophageal pressure and did not change significantly after laparotomy or post closure conditions under eupneic conditions. This suggests that negative intrathoracic pressure is maintained during open abdomen. However, under hypercapnic stress, we found that the changes in intrathoracic pressure were significantly greater compared to eupneic conditions. Pierce et al. described an increase in intrathoracic pressure under hypercapnic challenge in rat model (Pierce et al. 1998). In our experiment, we found that delta PES increased during all hypercapnic trials in comparison to eupnea, which was a reflection of increased overall inspiratory activity under chemical loading. However, even under hypercapnia, there were no significant differences in esophageal pressure between open and intact abdomen.

Our third hypothesis, that hypercapnia augments PIIA, was confirmed during intact abdominal conditions (Table 5). Under eupneic conditions, PIIA was augmented during open abdomen. However, the reverse trend was observed during hypercapnia where PIIA area/time ratios were significantly higher during intact abdomen (Table 2). Previously, Oliven et al. showed in dogs, hypercapnia induced progressive shortening of PIIA time in diaphragm and PS (Oliven et al. 1985). They also found under hypercapnia an increase in the activities of expiratory muscles like internal intercostal and external oblique, which may have countered the inspiratory muscle activity. Our data did not show similar results during intact abdomen conditions. However, during open and closed abdomen, PIIA was decreased under hypercapnia, consistent with finding of Oliven et al. It is also reasoned that PIIA is inversely affected by hypercapnia due to increased respiratory rate resulting in a decrease in expiratory time. However, we did not find any correlation between respiratory rate and PIIA time during hypercapnia and future studies may be necessary to investigate this component of the compensatory mechanism.

In addition to the changes in PIIA during hypercapnia, an increase in peak inspiratory muscle activity was observed, consistent with previous publications. Ainsworth et al. reported that hypercapnia augments mean EMG activity of the diaphragm in awake dogs (Ainsworth et al. 1989). Our results also showed during hypercapnia, the peak EMG amplitude was significantly higher in all inspiratory muscles compared to corresponding eupneic trials (Table 5). This suggest that with chemical loading, the respiratory pattern generator increases inspiratory muscle activity, which leads to increased peak amplitude, a reflection of increased inspiratory muscle activities.

Sedation and tracheostomy were essential to perform the study experiments, and of course, both interventions can potentially affect control of breathing and respiratory muscle recruitment. However, since all animals were tracheostomized and anesthetized during all experimental conditions (intact abdomen, open abdomen and post closed abdomen), changes in PIIA due to these conditions should not be affected by the tracheostomy and anesthesia and therefore both could not be considered confounding variables. Moderate sedation was chosen over deep sedation as it suppresses cortical responses, but keeps brain stem and spinal cord reflexes that affect PIIA intact. Placement of electrode in the diaphragm with abdomen intact is a blind procedure and often results in poor signal quality that could not be analyzed. Also, the risk of pneumothorax is involved, which may lead to postponement of the experiment even before laparotomy. This resulted in fewer numbers of prelaparotomy trials. Moreover, quality of EMG activity was used to provide indications that probes were properly positioned in the intended muscle groups. However, there is no guarantee that probes could be placed in the same spot within these muscles in all animals. This inconsistency creates variability in signal strength between the different animals, but it does not affect the study conclusions since it is the consistency in muscle activity measured at same spot during the different experimental condition in each individual animal was the most important (intrasubject vs. intersubject variability). Gastric pressure measurements were not obtained in this study, since assessing the effect of laparotomy on transdiaphragmatic pressure was not the principal objective. Instead, only esophageal pressure measurements were obtained for defining different phases of the respiratory cycles. Also, since volume loss is the main end result of laparotomy and lung volume is directly affected by pleural pressure more than by transdiaphragmatic pressure, we limited our measurements to esophageal pressure only.

In summary, our study showed that under eupnea PIIA is an important compensatory mechanism to counter mechanical instability induced by laparotomy. However, under hypercapnic trial, PIIA is shortened after laparotomy, possibly due to the increased respiratory rate and under the influence of increased expiratory muscle activity.

## Conflict of Interest

None declared.

## References

[phy212668-bib-0001] Ainsworth, D. M. , C. A. Smith , S. W. Eicker , K. S. Henderson , and J. A. Dempsey . 1989 The effects of chemical versus locomotory stimuli on respiratory muscle activity in the awake dog. Respir. Physiol. 78:163–176. 2609026 10.1016/0034-5687(89)90049-2

[phy212668-bib-0002] Basmajian, J. V. , and G. Stecko . 1962 A new bipolar electrode for electromyography. J. Appl. Physiol. 17:849–849.

[phy212668-bib-0003] Beecher, H. K. 1933a Effect of laparotomy on lung volume. Demonstration of a new type of pulmonary collapse. J. Clin. Invest. 12:651. 16694151 10.1172/JCI100526 PMC435931

[phy212668-bib-0004] Beecher, H. K. 1933b The measured effect of laparotomy on the respiration. J. Clin. Invest. 12:639. 16694150 10.1172/JCI100525 PMC435930

[phy212668-bib-0005] Burgess, K. , W. Whitelaw , and G. Ford . 1984 Diaphragm function and respiratory response after upper abdominal surgery in dogs. J. Appl. Physiol. 57:576–582. 6469824 10.1152/jappl.1984.57.2.576

[phy212668-bib-0006] Citterio, G. , and E. Agostoni . 1981 Decay of inspiratory muscle activity and breath timing in man. Respir. Physiol. 43:117–132. 7244430 10.1016/0034-5687(81)90004-9

[phy212668-bib-0007] Easton, P. A. , J.‐W. Fitting , R. Arnoux , A. Guerraty , and A. E. Grassino . 1989 Recovery of diaphragm function after laparotomy and chronic sonomicrometer implantation. J. Appl. Physiol. 66:613–621. 2708192 10.1152/jappl.1989.66.2.613

[phy212668-bib-0008] Easton, P. A. , H. G. Hawes , B. Rothwell , and A. De Troyer . 1999 Postinspiratory activity of the parasternal and external intercostal muscles in awake canines. J. Appl. Physiol. 87:1097–1101. 10484582 10.1152/jappl.1999.87.3.1097

[phy212668-bib-0009] Ford, G. , W. Whitelaw , T. Rosenal , P. Cruse , and C. A. Guenter . 1983 Diaphragm function after upper abdominal surgery in humans. Am. Rev. Respir. Dis. 127:431–436. 6838049 10.1164/arrd.1983.127.4.431

[phy212668-bib-0010] Gandevia, S. C. , R. B. Gorman , D. K. McKenzie , and A. De Troyer . 1999 Effects of increased ventilatory drive on motor unit firing rates in human inspiratory muscles. Am. J. Respir. Crit. Care Med. 160:1598–1603. 10556127 10.1164/ajrccm.160.5.9904023

[phy212668-bib-0011] Gautier, H. , J. Remmers , and D. Bartlett . 1973 Control of the duration of expiration. Respir. Physiol. 18:205–221. 4269825 10.1016/0034-5687(73)90051-0

[phy212668-bib-0012] Kosch, P. C. , A. Hutchinson , J. A. Wozniak , W. A. Carlo , and A. Stark . 1988 Posterior cricoarytenoid and diaphragm activities during tidal breathing in neonates. J. Appl. Physiol. 64:1968–1978. 3391897 10.1152/jappl.1988.64.5.1968

[phy212668-bib-0013] Lee, W. E. , G. Tucker , and L. Clerf . 1928 Post‐operative pulmonary atelectasis. Ann. Surg. 88:6. 17865923 10.1097/00000658-192807000-00002 PMC1398585

[phy212668-bib-0014] Oliven, A. , E. C. Deal , S. G. Kelsen , and N. S. Cherniack . 1985 Effects of hypercapnia on inspiratory and expiratory muscle activity during expiration. J. Appl. Physiol. 59:1560–1565. 2933384 10.1152/jappl.1985.59.5.1560

[phy212668-bib-0015] Panitch, H. B. 2015 Pulmonary complications of abdominal wall defects. Paediatr. Respir. Rev. 16:11–17. 25458796 10.1016/j.prrv.2014.10.004

[phy212668-bib-0016] Pierce, J. , R. Clancy , J. Trank , and J. Burris . 1998 Diaphragm shortening and intrathoracic pressure during hypercapnia in rats. Respir. Med. 92:4–8. 9519216 10.1016/s0954-6111(98)90023-3

[phy212668-bib-0017] Remmers, J. , and D. Bartlett . 1977 Reflex control of expiratory airflow and duration. J. Appl. Physiol. 42:80–87. 137890 10.1152/jappl.1977.42.1.80

[phy212668-bib-0018] Richter, D. 1982 Generation and maintenance of the respiratory rhythm. J. Exp. Biol. 100:93–107. 6757372 10.1242/jeb.100.1.93

[phy212668-bib-0019] Strauss‐Blasche, G. , M. Moser , M. Voica , D. McLeod , N. Klammer , and W. Marktl . 2000 Relative timing of inspiration and expiration affects respiratory sinus arrhythmia. Clin. Exp. Pharmacol. Physiol. 27:601–606. 10901389 10.1046/j.1440-1681.2000.03306.x

